# Dyadic effects of financial toxicity and social support on the fear of cancer recurrence in breast cancer patients and caregivers: an actor–partner interdependence mediation model

**DOI:** 10.1186/s12912-024-02046-0

**Published:** 2024-06-05

**Authors:** Hongyan Li, Yabin Sun, Tianye Yang, Xin Yin, Zhu Zhu, Jianjun Shi, Lingling Tong, Jia Yang, Hui Ren

**Affiliations:** 1 The First Hospital of Jilin University, Changchun, Jilin Province China; 2 Heping Hospital Affiliated to Changzhi Medical College, Changzhi, Shanxi Province China; 3 China-Japan Union Hospital of Jilin University, Changchun, Jilin Province China; 4 Changchun Central Hospital, Changchun, Jilin Province China

**Keywords:** Breast cancer, Fear of cancer recurrence, Financial toxicity, Social support, Actor–partner interdependence mediation model

## Abstract

**Purpose:**

In this study, the actor–partner interdependence mediation model (APIMeM) was applied to breast cancer patients and their caregivers to assess the factors that affect the fear of cancer recurrence. In particular, the purpose of this study was to evaluate the mediating effect of social support on financial toxicity and the fear of cancer recurrence, providing an effective basis for developing plans to reduce the level of fear of cancer recurrence.

**Methods:**

This study employed a cross-sectional design, and 405 dyads of breast cancer patients and their caregivers were enrolled. Financial toxicity, social support, and fear of cancer recurrence were assessed by computing comprehensive scores for financial toxicity based on patient-reported outcome measures, the Social Support Rating Scale, and the Fear of Cancer Recurrence Inventory Short Form, respectively. The data were analysed using SPSS 24.0 and AMOS 23.0.

**Results:**

The results showed that the fear of cancer recurrence of breast cancer patients and their caregivers was significantly related to dyadic financial toxicity and social support. In addition, the financial toxicity of breast cancer patients and their caregivers had significant actor effects and partner effects on the fear of cancer recurrence through dyadic social support.

**Conclusions:**

The financial toxicity of breast cancer patients and their caregivers could produce actor and partner effects on the fear of cancer recurrence through the mediation of social support, which provided empirical support for improving reducing the level of fear of cancer recurrence among patients and caregivers at the dyadic level.

## Background

In China, breast cancer ranks first and fourth in incidence and mortality, respectively, among female patients with malignant tumours, and the numbers of cases and deaths continue to rise [1]. The 5-year survival rate of patients with breast cancer continues to improve with the progress of early diagnosis, early treatment and medical treatment [1]. However, because of the pathophysiological characteristics of breast cancer, recurrence and metastasis are still the greatest problems faced by patients [1]. Therefore, breast cancer patients are commonly fearful of cancer recurrence [2, 3] during long-term treatment and throughout life [2, 3]. This “mental state of fear or worry that cancer may recur or progress” is called the fear of cancer recurrence (FCR) [4]. Research has shown that [5, 6] the incidence of FCR in breast cancer patients is 42-70%. High levels of FCR increase the incidence of anxiety and depression in patients and impair their quality of life [7]. Therefore, the FCR of breast cancer patients warrants further attention.

In addition, breast cancer patients and caregivers are interdependent when dealing with cancer because the diagnosis and treatment of cancer not only cause severe psychological pain to patients but also affect the mental health of caregivers. Caregivers must not only bear the economic pressure and responsibility of taking care of patients but also endure the torment of patients’ fears over a long period of time and are accordingly affected by FCR [8]. Research has shown that [9] 51.6% of breast cancer patient caregivers had a high level of FCR, and the degree of FCR was greater than that of patients [10]. Caregivers’ high FCR can not only increase the psychological distress of patients but may also affect the treatment and recovery of patients’ diseases. However, at present, breast cancer patient and caregivers FCR has received insufficient attention.

Financial toxicity refers to the negative impact of financial expenditures on cancer patients and their families during the treatment process [11]. Financial toxicity is common in cancer patients and is a common source of psychological stress for both patients and family caregivers during cancer treatment [12]. Previous studies have shown that financial difficulties are associated with more severe psychological symptoms, such as anxiety and depression, which can place a greater burden on patients and their caregivers [13, 14]. According to a literature review, some scholars have focused on the relationship between financial conditions and FCR, believing that financial conditions can increase FCR in patients or caregivers to a certain extent [15–17]. However, at present, some researchers have analysed the relationship between financial toxicity and FCR at the individual level, while the role of financial toxicity on caregiver FCR in breast cancer patients and caregivers remains unclear and must be discussed further.

The social cognitive processing model emphasizes that a supportive social environment helps patients engage in good cognitive processing, thereby improving their psychological and social adaptability [18]. For example, a meta-analysis confirmed that social support that encourages emotional expression can improve patients’ painful emotions and FCR [19]. In addition, some studies have shown that support from family, friends, and health care professionals can effectively help patients alleviate stress and cope with uncertainty, thus helping to control their FCR [20, 21]. Social support is an important way to improve the prevalence of FCR. Research has confirmed that the social support of cancer patients is related to their treatment outcomes [22]. When patients face financial toxicity issues, they usually choose to reduce daily expenses, change their existing lifestyle habits, reduce social interaction activities etc., to cope with the high economic burden of cancer treatment [17, 23]. In addition, varying degrees of negative emotions may exist, such as anxiety, worry, and high levels of perceived stress [14]. If effective and objective social resource support and subjective emotional support can be obtained in a timely manner, these measures will be beneficial for ameliorating health conditions and negative emotions. Therefore, it is necessary to clarify the role of social support in the relationship between financial toxicity and FCR in breast cancer patients and caregivers.

The theory of stress and coping [24] suggests that when an individual experiences a stressful event, they appraise and cope with it before choosing the best coping method, which may have a potential impact on the outcome. In this study, the stressful event was financial toxicity, and patients experienced an increase in treatment expenses and an objective burden, which had an impact on subjective distress. Appraisal and coping are cognitive processes in which individuals reflect on the outcomes of stressful events and expectations. Individuals respond to stressful events based on their interactions with their environment [25]. Discussing stressful events in a supportive social environment can help individuals engage in good cognitive processing, thereby improving their psychological adaptation process. In contrast, public or covert restrictive behaviours in the social environment can cause patients to avoid thinking about or discussing stressful events, leading to increased psychological stress and impaired psychological adaptability [26]. This result is the outcome of the assessment and coping process of social support impact. *I*n this study, FCR was used as the outcome variable to explore the impact of financial toxicity on FCR from economic and social cognitive dimensions. Assuming that when individuals face financial toxicity, they may be unable to participate in social activities and lose social relationships, both of which have negatives impact on fear of cancer recurrence.

Although the financial toxicity and social support of breast cancer patients and their caregivers are the main factors that affect FCR, many studies have focused solely on patients. This approach makes the establishment of effective interventions for FCR difficult. The relationships between patients and their caregivers are complementary and include sharing and solving daily problems. Accordingly, the psychological issues of patients should not be studied separately. In summary, the aim of this study was to explore the dyadic effects of financial toxicity and social support on FCR in breast cancer patient–caregiver dyads.

## Methods

### Participants

A cross-sectional design was employed in this study. From June 2020 to January 2021, breast cancer patients and their caregivers from four Grade-A general hospitals in China were selected for on-site and online surveys.

The inclusion criteria for patients were as follows: (1) aged 18 to 80 years; (2) who had been diagnosed with primary breast cancer and had undergone surgery to remove cancer tissue, and the postoperative time was less than 5 years; (3) had the ability to understand Chinese questionnaires and communicate in the language; and (4) voluntary participation and willingness to sign an informed consent form. The exclusion criteria were as follows: (1) did not know of their own diagnosis of breast cancer; (2) were suspected of having recurrence or metastasis or had already experienced metastasis or recurrence; (3) were excessively weak and unable to cooperate with this survey; and (4) had a cognitive impairment, mental illness, or previous psychological therapy. The inclusion criteria for breast cancer caregivers were as follows: (1) aged 18 years or older and responsible for primary care for the breast cancer patient; (2) able to communicate and interact normally, with normal language expression and comprehension abilities; and (3) voluntary participation in this study. The exclusion criteria were caregivers who (1) had serious physical illnesses or (2) had a cognitive impairment or mental illness.

During the survey period of this study, 447 pairs of breast cancer patients and their caregivers were sent questionnaires, 42 pairs of unqualified questionnaires were excluded, and 405 pairs of effective questionnaires were returned, for an effective recovery rate of 90.60%. This study was approved by the Ethics Committee of the School of Public Health, Jilin University.

### Measures

#### Fear of Cancer Recurrence Inventory Short Form (FCRI-SF)

This study used the FCRI-SF to measure the level of FCR. This instrument assesses the severity of invasive thoughts and the perceived risks associated with FCR in breast cancer patients [27]. The FCRI-SF contains 9 items, of which is each rated on a 5-point Likert scale ranging from 0 (not at all or never) to 4 (very much or all the time), for a total possible score of 0–36 points. The respondents were classified as having a clinical level of FCR if their score was 13 or higher. The higher the score, the higher the level of FCR of the research subject. Cronbach’s α of this study was 0.88.

#### Comprehensive scores for financial toxicity based on patient-reported outcome measures (COST-PROM)

This study used COST-PROM [28], which was constructed by Souza et al. at the University of Chicago in 2014. The purpose of this instrument was to assess the subjective and objective financial and job-related stress perceived by cancer patients. The scale consists of 11 items and 3 dimensions (financial expenditure, financial resources, and psycho-social responses). A 5-point Likert scale ranging from 0 (not at all) to 4 (very) was used, with a total possible score ranging from 0 to 44; as the lower the score is, the more severe the financial difficulties faced by the participants. The scale has been verified by multiple studies to have high reliability and validity [29, 30]. Cronbach’s α of this study was 0.87.

#### Social Support Rating Scale (SSRS)

This study used the SSRS developed by Xiao Shuiyuan [31] to measure the social support level of the study participants. The scale consists of 10 items and 3 dimensions: objective support, subjective support, and utilization of support. Items 1–5 and 8–10 are rated on a 4-point Likert scale ranging from 1 (not at all) to 4 (very much). For Items 6 and 7, if “No source” is answered, a score of 0 is assigned; if “have a source” is answered, each source is assigned 1 point. The total SSRS score ranges from 12 to 66. The higher the score is, the greater the social support level of the research subject. Cronbach’s α of this study was 0.83.

### Statistical Methods

Lederman et al. [32] proposed the actor–partner interdependence mediation model (APIMeM), which suggested that dyad groups with intimate relationships, such as emotional cognition or behaviour, can influence each other as a result of interpersonal interactions. This model uses paired data analysis to compensate for the shortcomings of traditional methods and can systematically explain the complex relationship between patients and caregivers as interaction units in the cancer adaptation process. This model not only reflects the impact of individual traits on oneself but also evaluates the mutual influence with caregivers, including both actor and partner effects. The actor effect refers to the effect of an individual’s independent variable on their own dependent variable, while the partner effect refers to the effect of an individual’s independent variable on the partner’s dependent variable.

This study used SPSS 24.0 and AMOS 23.0 software (IBM Corporation, Armonk, New York, USA) to analyse the data. Two-sided significance tests were performed at *P* ≤ 0.05. Descriptive statistics were generated as the means and standard deviations for continuous variables, as well as proportions and counts for categorical variables. Pearson’s correlation analyses were conducted to investigate the relationships between financial toxicity, social support and FCR in patient-caregiver dyads. APIMeM was conducted to examine the relationships between financial toxicity experienced by breast cancer patients and caregivers and FCR at the dyadic level, as well as the mediating role of social support. The effect size was estimated using the maximum likelihood method, and the confidence interval was estimated using Bootstrap sampling. The sample size was set to 5000, and the 95% confidence interval (CI) excluding 0 indicates the presence of mediation. Model fit [33]was evaluated by the goodness of fit indices, which included χ^2^/df (acceptable fit < 3), the Root Mean Square Error of Approximation (RMSEA, acceptable fit ≤ 0.08), the Comparative Fit Index (CFI, acceptable fit ≥ 0.90), the Tucker–Lewis index (TLI, acceptable fit ≥ 0.90), and the Normed Fit Index (NFI, acceptable fit ≥ 0.90).

## Results

### Subjects’ sociodemographic and clinical characteristics

In this study, a total of 810 participants were involved, comprising 405 patients and 405 caregivers. Table 1 provides a summary of the sociodemographic and clinical characteristics of the patient–caregiver dyads. In the present study, the FCR score of individuals diagnosed with breast cancer was 17.95 ± 9.61, while that of their spouses was 18.74 ± 9.81. Based on the established threshold score for mental dysfunction, which is defined as reaching the level of clinical significance, 219 breast cancer patients (54.1%) and 204 spouses (50.4%) exhibited a score exceeding 13 on the FCR scale. The average ages of the patients and caregivers were 49.32 ± 9.69 years and 42.28 ± 11.61 years, respectively. The majority of participants had a college education or a junior high school education or above. Additionally, more than half of the participants were of Han nationality. The majority of patients had stage II breast cancer, 83.7% of whom did not undergo breast conserving surgery. The auxiliary treatment methods mainly include preoperative neoadjuvant chemotherapy (136, 33.6%), postoperative adjuvant chemotherapy (196, 48.4%). Additionally, 111 patients have also received varying degrees of radiation therapy.

**Table 1:** Socio-demographic and clinical characteristics of patient-caregiver dyads

Variables		Patients	Caregivers
		(*n* = 405) N (%)/M ± SD	(*n* = 405) N (%)/M ± SD
Financial toxicity		17.95 ± 9.61	18.74 ± 9.81
Social support		33.88 ± 9.44	33.98 ± 9.46
Fear of cancer recurrence		15.61 ± 9.50	15.11 ± 9.28
Educational level	Primary school and below	50(12.4)	67(16.5)
	Junior high school	134(33.1)	130(32.1)
	High school	106(26.2)	90(22.2)
	University and college	115(28.3)	118(29.2)
Employment status	Employed	294(72.6)	311(76.8)
	Unemployed	111(27.4)	94(23.2)
Nationality	Han nationality	387(95.6)	380(93.8)
	Other nationalities	18(4.4)	25(6.2)
Chronic underlying diseases	No	305(75.3)	308(76.0)
	Yes	100(24.7)	97(24.0)
Staging of breast diseases	I	125(30.9)	
	II	201(49.6)	
	III	79(19.5)	
Breast conserving surgery	No	339(83.7)	
	Yes	66(16.3)	
Chemotherapy	No	73(18.0)	
	Neoadjuvant chemotherapy	136(33.6)	
	Postoperative adjuvant chemotherapy	196(48.4)	
Radiotherapy	No	294(72.6)	
	Yes	111(27.4)	

### Correlations between financial toxicity, social support and FCR

Correlation analyses revealed that patients’ and caregivers’ financial toxicity (*r* = 0.95, *P* < 0.01), social support (*r* = 0.94, *P* < 0.01) and FCR (*r* = 0.96, *P* < 0.01) were significantly positively related. Patients’ financial toxicity and was significantly negatively related with social support of patents (*r*=-0.91, *P* < 0.01) and caregivers (*r*=-0.91, *P* < 0.01), and significantly positively correlated with FCR (*r* = 0.94, *P* < 0.01) of patients and caregivers (*r* = 0.94, *P* < 0.01). Patients’ social support was significantly negatively related with FCR of patients (*r*=-0.92, *P* < 0.01) and caregivers (*r*=-0.91, *P* < 0.01), and significantly negatively related with financial toxicity of patients (*r*=-0.90, *P* < 0.01). Patients’ FCR was significantly positively related with financial toxicity (*r* = 0.93, *P* < 0.01) and significantly negatively related with social support of caregivers (*r*=-0.92, *P* < 0.01). Caregivers’ financial toxicity was significantly negatively related with support (*r*=-0.91, *P* < 0.01) and positively related with FCR (*r* = 0.93, *P* < 0.01). Caregivers’ social support was significantly negatively related with support (*r*=-0.91, *P* < 0.01).

### Actor–partner interdependence mediation model analysis

**Fig. 1:**
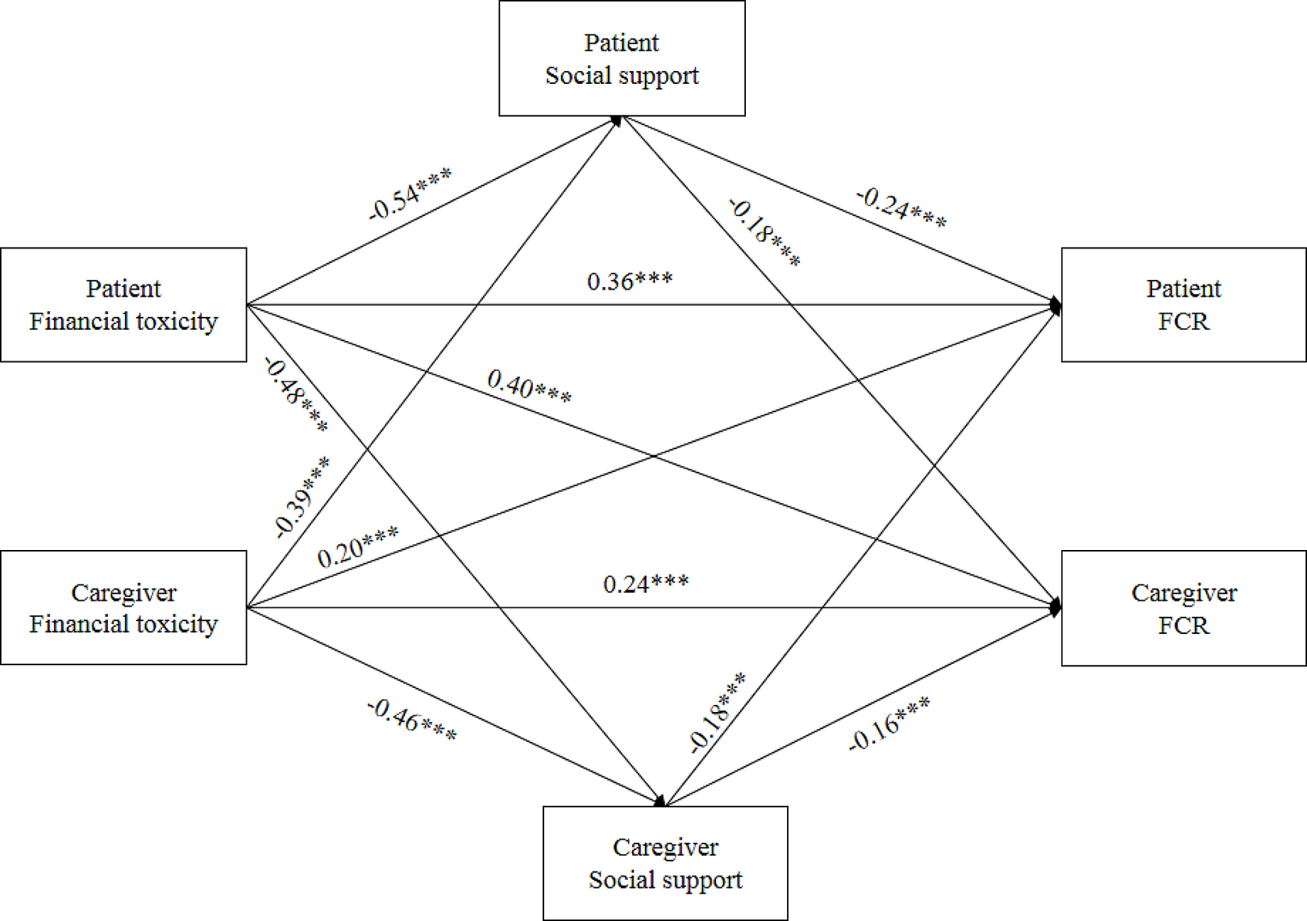
APIMeM results of financial toxicity, social support on FCR. *Note*. APIMeM: Actor-partner interdependence mediation model; FCR: fear of cancer recurrence. Values are standardized coefficients. ^***^p < 0.001

The APIMeM is displayed in Fig. 1. The final APIMeM model examining the dyadic effects of financial toxicity and social support on FCR produced a satisfactory model fit (χ^2^/df = 2.400, RMSEA = 0.059, CFI = 0.998, TLI = 0.996, NFI = 0.997). The results showed that the financial toxicity of patients had a negative actor effect on their own social support (*β*=-0.54, *P* < 0.001), and a negative partner effect on caregivers’ social support (*β*=-0.48, *P* < 0.001). The social support of patients had a negative actor effect on their own FCR (*β*=-0.24, *P* < 0.001), and a negative partner effect on caregivers’ FCR (*β*=-0.18, *P* < 0.001). The financial toxicity of patients had a positive actor effect on their own FCR (*β* = 0.36, *P* < 0.001), and a positive partner effect on caregivers’ FCR (*β* = 0.40, *P* < 0.001). The financial toxicity of caregivers had a negative actor effect on their own social support (*β*=-0.46, *P* < 0.001), and a negative partner effect on patients’ social support (*β*=-0.39, *P* < 0.001). The social support of caregivers had a negative actor effect on their own FCR (*β*=-0.16, *P* < 0.001), and a negative partner effect on patients’ FCR (*β*=-0.18, *P* < 0.001). The financial toxicity of caregivers had a positive actor effect on their own FCR (*β* = 0.24, *P* < 0.001), and a positive partner effect on patients’ FCR (*β* = 0.20, *P* < 0.001).

Through the bootstrap test, the results showed that in the relationship between the financial toxicity of breast cancer patients and caregivers and their own FCR, their own social support plays a positive mediating role (*β* = 0.130, *P* < 0.001, 95% CI = 0.078–0.192; *β* = 0.070, *P* < 0.001, 95% CI = 0.033–0.116); namely, the actor–actor effect is established. In the relationship between the financial toxicity of breast cancer patients and their caregivers and dyadic FCR, their own social support also plays a positive mediating role (*β* = 0.079, *P* < 0.001, 95% CI = 0.041–0.128; *β* = 0.092, *P* < 0.001, 95% CI = 0.046–0.150); namely, the actor–partner effect is significant. In the relationship between the financial toxicity of breast cancer patients and caregivers and their own FCR, the social support of the dyad played a positive mediating role (*β* = 0.084, *P* < 0.001, 95% CI = 0.040–0.137; *β* = 0.066, *P* < 0.001, 95% CI = 0.032–0.109); that is, the partner-partner effect was established. In terms of the relationship between the financial toxicity of breast cancer patients and their caregivers and dyadic FCR, dyadic social support also plays a positive mediating role (*β* = 0.093, *P* < 0.001, 95% CI = 0.056–0.137; *β* = 0.075, *P* < 0.001, 95% CI = 0.036–0.124); namely, the partner–actor effect is significant. The results of the direct effects and indirect effects of financial toxicity on FCR are displayed in Table 2.

**Table 2:** Standardized Total effects, Indirect effects, and direct effects of patients’ and caregivers’ financial toxicity on FCR via social support

Effect			Beta	*P*(two-tailed)	BC 95%CI
Actor effect (individual’s financial toxicity-individual’s FCR)					
Patient	Total effect		0.572	<0.001	(0.459–0.679)
	Total IE		0.214	< 0.001	(0.160–0.278)
	Actor-actor simple IE	Patient financial toxicity-patient social support-patient FCR	0.130	< 0.001	(0.078–0.192)
	Partner-partner simple IE	Patient financial toxicity-caregiver social support-patient FCR	0.084	< 0.001	(0.040–0.137)
	Direct effect	Patient financial toxicity-patient FCR	0.358	< 0.001	(0.256–0.460)
Caregiver	Total effect		0.364	< 0.001	(0.261–0.463)
	Total IE		0.136	< 0.001	(0.097–0.183)
	Actor-actor simple IE	Caregiver financial toxicity-caregiver social support-caregiver FCR	0.070	< 0.001	(0.033–0.116)
	Partner-partner simple IE	Caregiver financial toxicity-patient social support-caregiver FCR	0.066	< 0.001	(0.032–0.109)
	Direct effect	Caregiver financial toxicity-caregiver FCR	0.227	< 0.001	(0.129–0.330)
Partner effect (individual’s financial toxicity-partner’s FCR)					
Patient	Total effect		0.368	< 0.001	(0.262–0.478)
	Total IE		0.171	< 0.001	(0.125–0.225)
	Actor-partner simple IE	Caregiver financial toxicity-caregiver social support-patient FCR	0.079	< 0.001	(0.041–0.128)
	Partner-actor simple IE	Caregiver financial toxicity-patient social support-patient FCR	0.093	< 0.001	(0.056–0.137)
	Direct effect	Caregiver financial toxicity-patient FCR	0.197	< 0.001	(0.097–0.297)
Caregiver	Total effect		0.555	< 0.001	(0.456–0.659)
	Total IE		0.167	< 0.001	(0.122–0.227)
	Actor-partner simple IE	Patient financial toxicity-patient social support-caregiver FCR	0.092	< 0.001	(0.046–0.150)
	Partner-actor simple IE	Patient financial toxicity-caregiver social support-caregiver FCR	0.075	< 0.001	(0.036–0.124)
	Direct effect	Patient financial toxicity-caregiver FCR	0.387	< 0.001	(0.293–0.484)

Note. IE: indirect effect; CI: confidence interval; FCR: fear of cancer recurrence

## Discussion

Most previous studies focused only on the actor effect between financial toxicity and FCR. Therefore, in order to supplement the existing research, this study attempted to assess the effects of financial toxicity on FCR through social support by measuring a dyadic approach. The results of the study revealed that the financial toxicity of breast cancer patients and their caregivers had significant actor effects and partner effects on FCR through social support.

In this study, 405 pairs of breast cancer patients and their caregivers were investigated. The FCR score of the patient was determined to be 17.95 ± 9.61, with 54.1% of the patients reaching the clinically significant threshold. The FCR score of the caregivers was 18.74 ± 9.81, 50.4% of whom exceeded the clinically significant level. These results are consistent with those of previous studies [10, 34], indicating that FCR is a common psychological problem among breast cancer patients and their caregivers, thus emphasizing the importance of solving this problem. In addition, it was observed that the FCR levels of their caregivers were significantly higher than those of the patients themselves. These findings are consistent with the conclusions drawn by Xu et al. [34]. Caregivers are not only responsible for providing lasting emotional and economic support, as well as daily care, but also for facing the uncertainty of the disease. Therefore, this situation often results in a psychological burden and varying degrees of FCR, with the severity of FCR even exceeding what the patient has experienced themselves. At the same time, we found that 20% of patients had stage III disease, which might have impacted the results of financial toxicity and FCR. Patients with advanced breast cancer need more extensive surgery and longer treatment times, and subsequent treatment and complications can affect their emotions [29], increasing their level of FCR.

The results of this study showed that the financial toxicity level of breast cancer patients and their caregivers can not only positively predict their own FCR but also positively predict each other’s FCR; namely, the actor–partner effect was valid. According to a literature review, no research has explored the actor–partner effects of financial toxicity of breast cancer patients and their caregivers on FCR, and this study is a useful supplement to related fields. When facing financial difficulties, breast cancer patients and their caregivers have to bear the dual pressure of the pain of the disease itself and medical expenses; they are concerned that the enormous cost of cancer recurrence will lead to further poverty in their families, and their FCR is more pronounced [14, 35].

This study revealed a negative correlation and actor–partner effect between social support and FCR in patients and caregivers. Social support mainly refers to the behavior of using spiritual and material means to help vulnerable groups in society, which is an important buffering factor in the face of stressful events for individuals [36, 37]. Social support can not only protect individuals from harm but also help them alleviate negative emotions, enhance confidence in disease treatment and rehabilitation, improve treatment compliance, and reduce FCR [19, 38]. However, at present, research on the relationship between FCR and social support is limited, especially among caregivers of breast cancer patients. This study is a useful supplement to this field. Therefore, in future research, we should devote further attention to the role of social support to alleviate the FCR of patients and their caregivers and improve their mental health.

Consistent with prior work [39], this study revealed that financial toxicity had negative actor and partner effects on social support in patients and caregivers. The buffer theory of social support suggests that when an individual experiences stressful events, social support can play a certain buffering role [40]. Social support reduces negative emotions caused by financial toxicity events by providing material, emotional, and informational support to patients, thereby improving the individual’s health status [41]. In addition, this study revealed that 24% of patients have chronic underlying diseases, which may affect the results. When patients face the dual economic burden of chronic diseases and breast cancer, they have a high level of financial toxicity. These patients experience various adverse symptoms, leading to a fragile immune system and impaired physical image, which makes it difficult for them to maintain close contact with people [42]. In addition, the treatment process is usually lengthy and time-consuming, leading to a lack of necessary social interaction, which may weaken their social support network and lead to a decrease in their social support level [29]. Thus, the findings of this study expand existing research on the relationship between financial toxicity and the social support of breast cancer patients and caregivers in China.

The mediation analysis results showed that patients’ financial toxicity influence their FCR through the mediation effect of perceived social support. Social support is the material or spiritual assistance provided by social groups, relatives, friends, etc. When patients face financial toxicity issues, they usually choose positive coping strategies to cope with the high economic burden of cancer treatment. In addition, varying degrees of negative emotions may be present. If effective objective social resource support and subjective emotional support can be obtained in a timely manner, they will assist in reducing the level of FCR and improve their health conditions. In addition, this study revealed that the financial toxicity of breast cancer patients and their caregivers could affect dyadic FCR through dyadic social support, which was another important finding of this study. This relationship demonstrated the necessity of exploring the relationships among financial toxicity, social support and FCR at the dyad level. As the main body of the whole dyad, breast cancer patients and their caregivers are the main sources of mutual emotional support, and both parties should jointly address the disease. When individuals face financial toxicity, high level of social support helps them express their thoughts during their cancer experiences, encourages them to find solutions, and reduces dyadic fears. This result suggested that future research can implement targeted intervention measures to reduce the financial toxicity level of breast cancer patients and their caregivers from a dyad perspective, provide some social support, and alleviate the FCR level on both sides.

Some limitations should be acknowledged. First, this was a cross-sectional study, and thus, establishing a causal relationship is impossible. In the future, a longitudinal research design can be adopted to further explore the dynamic trends and relationships between variables. Although the sample in this study came from four hospitals, multi-centre large-scale surveys should be conducted to further verify the results of the study in the future. The research data were obtained from patients and caregivers through self-reports questionnaires, which may have led to reporting bias and social expectation bias. Finally, this study used observed rather than latent variables, and future research on latent variables should be strengthened to make the research more targeted.

## Conclusions

The financial toxicity of breast cancer patients and their caregivers can produce actor and partner effects on FCR through the mediation of social support, which provides empirical support for ameliorating the FCR of patients and caregivers at the dyad level.

This finding suggests that future research should focus on the financial toxicity level of breast cancer patients and their caregivers at the dyadic level, fully consider and address questions from both sides during disease treatment, provide social support, alleviate the dyadic psychological burden and improve the FCR level.

## Data Availability

The datasets used and/or analysed during the current study are available from the corresponding author on reasonable request.
